# Specificity of the Metallothionein-1 Response by Cadmium-Exposed Normal Human Urothelial Cells

**DOI:** 10.3390/ijms20061344

**Published:** 2019-03-17

**Authors:** Rhiannon V. McNeill, Andrew S. Mason, Mark E. Hodson, James W.F. Catto, Jennifer Southgate

**Affiliations:** 1 Jack Birch Unit for Molecular Carcinogenesis, Department of Biology, York Biomedical Research Institute, University of York, York YO10 5DD, UK; rvm8828@gmail.com (R.V.M.); andrew.mason@york.ac.uk (A.S.M.); 2 Department of Environment and Geography, University of York, York YO10 5DD, UK; mark.hodson@york.ac.uk; 3 Academic Urology Unit, University of Sheffield, Sheffield S10 2TN, UK; j.catto@sheffield.ac.uk

**Keywords:** Metallothionein, urothelium, urothelial cancer, cadmium exposure, zinc transporter

## Abstract

Occupational and environmental exposure to cadmium is associated with the development of urothelial cancer. The metallothionein (MT) family of genes encodes proteins that sequester metal ions and modulate physiological processes, including zinc homeostasis. Little is known about the selectivity of expression of the different MT isoforms. Here, we examined the effect of cadmium exposure on MT gene and isoform expression by normal human urothelial (NHU) cell cultures. Baseline and cadmium-induced MT gene expression was characterized by next-generation sequencing and RT-PCR; protein expression was assessed by Western blotting using isoform-specific antibodies. Expression of the zinc transporter-1 (*SLC30A1*) gene was also assessed. NHU cells displayed transcription of *MT-2A*, but neither *MT-3* nor *MT-4* genes. Most striking was a highly inducer-specific expression of MT-1 genes, with cadmium inducing transcription of *MT-1A*, *MT-1G*, *MT-1H*, and *MT-1M*. Whereas *MT-1G* was also induced by zinc and nickel ions and *MT-1H* by iron, both *MT-1*A and *MT-1M* were highly cadmium-specific, which was confirmed for protein using isoform-specific antibodies. Protein but not transcript endured post-exposure, probably reflecting sequestration. *SLC30A1* transcription was also affected by cadmium ion exposure, potentially reflecting perturbation of intracellular zinc homeostasis. We conclude that human urothelium displays a highly inductive profile of MT-1 gene expression, with two isoforms identified as highly specific to cadmium, providing candidate transcript and long-lived protein biomarkers of cadmium exposure.

## 1. Introduction

Occupational and environmental exposure to cadmium has increased as a result of the burning of fossil fuels and widespread use of the “heavy” metal in anthropological activities, such as battery production, electroplating, smelting, and soldering (reviewed [1]). Cadmium ions accumulate in the body in an almost irreversible manner [2], as the metal cannot be metabolized to a less toxic species [3] and has a low excretion rate [4]. This low excretion rate is thought to be due to intracellular sequestration of cadmium ions by metal-binding proteins [5,6,7]. An association between cadmium exposure and bladder (urothelial) carcinogenesis has been reported, with higher cadmium concentrations demonstrated in the blood [8] and urine [9,10,11] of patients with bladder cancer. In vitro research supports these correlative studies, with malignant transformation of the immortalized RWPE-1, TLR1215, 16HBE, and UROtsa cell lines reported after extended chronic cadmium exposure [12,13,14,15]. The bladder stores concentrated urine prior to voiding, meaning that the urothelial lining of the bladder (which functions as one of the tightest epithelial barriers [16]) is potentially exposed to excreted xenobiotics [16,17,18]. It is currently not known whether exposure to urinary cadmium is limited by the presence of an intact urinary barrier.

The metallothioneins (MTs) are a superfamily of low-molecular weight (~6 kDa), cysteine-rich proteins that are induced by and bind a range of metal ions, including cadmium [19,20]. Through this sequestration of metal ions MTs are considered to play a primary role in metal detoxification [21,22], but also metal (e.g., zinc) homeostasis [23,24,25] and the scavenging of reactive oxygen species (ROS) [26,27]. MT involvement in the bodily response to cadmium exposure has been well-documented, with numerous studies demonstrating cadmium-induced MT expression both in vitro [28,29,30,31,32,33,34] and in vivo [35,36].

Direct binding of MT protein to cadmium ions [37,38] results in a MT-cadmium complex that is highly resistant to degradation [5,6,7]. In humans, four main MT subfamilies exist (MT-1, MT-2/2A, MT-3, and MT-4), with MT-1 consisting of nine isoforms (-A, -B, -E, -F, -G, -H, -L, -M, and -X) [39] each encoded on individual genes. It is predicted that the individual MT isoforms have distinct properties including structure [40,41], tissue- and inducer-specific expression [22,34,42,43,44,45,46,47], induction rate [48], translational efficiency [41], and degradation rate [49,50]. Cadmium is reported to be the most potent inducer of MT expression [51]. This offers the potential that individual MT isoforms may be utilized as specific biomarkers of human exposure to cadmium, although it remains unclear which isoform(s) are responsible for cadmium sequestration. This lack of discrimination is largely due to the high homology between isoforms and the lack of discriminatory reagents, with no validated antibodies able to distinguish MT-1 and MT-2 subfamilies, nor the different MT-1 isoforms [41,52].

MTs are reported to work cooperatively with zinc transporters to regulate cellular zinc homeostasis, potentially by modulating cellular zinc ion concentration [39], although the exact mechanisms are unknown. Thus, a possible consequence of exposure to cadmium ions may be altered cellular zinc homeostasis. Cadmium and zinc possess highly similar properties, and it has been shown that cadmium can substitute for zinc in biological systems [53]. This can disrupt the normal functioning of various biological pathways [54,55], thus indirectly influencing processes involved in carcinogenesis such as cell proliferation and metastasis [56].

Our aims were to investigate MT isoform expression and specificity of induction in human urothelium under baseline and cadmium-exposed conditions, using a well-characterized normal human urothelial (NHU) cell culture system that includes polarized differentiated NHU cell sheets possessing tight barrier function [57,58]. Prior to cadmium exposure, cellular growth assays were performed to assess cytotoxicity. Next, we exposed NHU cells to a variety of potential MT inducers, including reactive oxygen species (ROS) [59,60], essential metals [44,61,62,63,64,65,66], and heavy metals [67,68,69,70] to define the specificity of response. Previously unpublished MT-1 isoform-specific antibodies were used to discriminate between MT-1 isoform proteins. Lastly, we determined whether transcription of the free zinc efflux regulator zinc transporter-1 (*SLC30A1*) [71] was altered as a consequence of cadmium exposure. The results revealed that MT isoform expression was inducer-specific, and that abundance of both *MT-1A* and *MT-1M* transcript and protein was highly cadmium-specific, highlighting their potential as biomarkers of exposure. Cadmium was able to penetrate an intact urothelial barrier and effected transcriptional upregulation of *SLC30A1*, indicating a potential route for cadmium uptake and possible subsequent substitution in zinc homeostatic mechanisms.

## 2. Results

### 2.1. Influence of Cadmium on NHU Cell Culture Growth and Uptake of Cadmium across an Intact Urothelial Barrier

**Figure 1:**
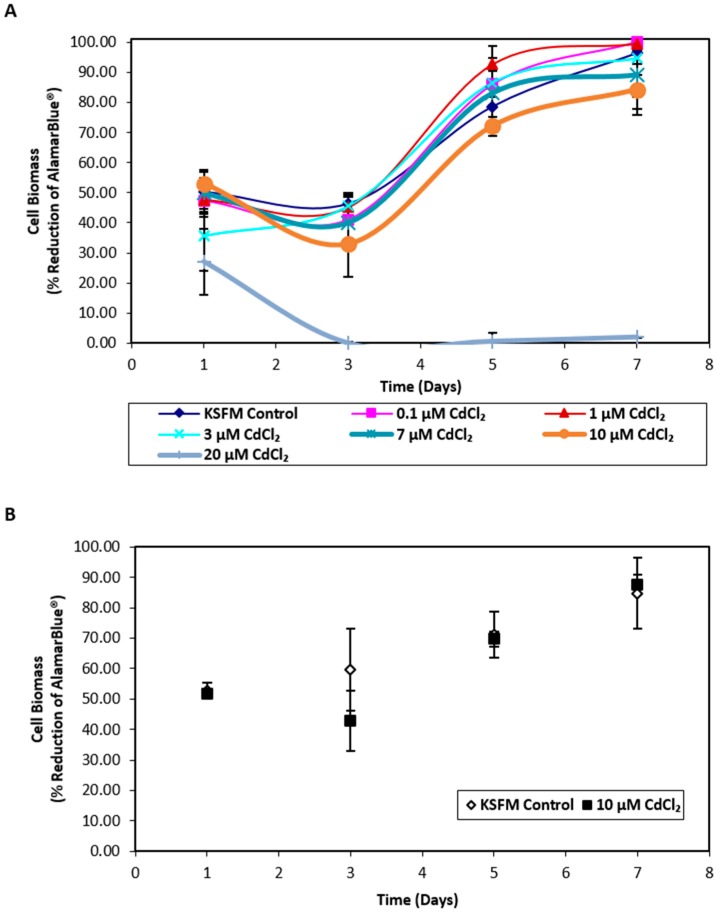
Biomass growth assays for in vitro normal human urothelial (NHU) cell cultures exposed to cadmium. AlamarBlue^®^ assays were performed over 7 days on NHU cell cultures seeded at 6 × 10^4^ cells/cm^2^. (**A**) NHU cells were exposed to a range of cadmium concentrations from 0 to 20 µM (*n* = 1 independent cell line). Each data point represents mean percentage reduction in AlamarBlue^®^ ± S.D. from three replicate cultures. (**B**) NHU cells were exposed to 10 µM CdCl_2_ for up to 7 days. Data points represent mean percentage reduction in AlamarBlue^®^ ± S.D. from two independent NHU cell lines, each performed in triplicate.

Exposure of nondifferentiated NHU cells to cadmium revealed that cell growth was unaffected by concentrations ≤ 10 µM CdCl_2_ whilst exposure to 20 µM CdCl_2_ resulted in distinct cytotoxicity (Figure 1A). Replication in a second independent cell line confirmed that exposure to 10 µM CdCl_2_ did not affect NHU cell growth (Figure 1B); this concentration was therefore selected for further experiments. When differentiated NHU cell cultures (three independent cell lines) were grown on permeable membranes in triplicate and exposed apically to 10 µM CdCl_2_ for 72 h, no effect was seen on barrier function (control versus cadmium-exposed transepithelial electrical resistance (TEER) of 3.24 ± 0.48 kΩ.cm^2^ versus 3.17 ± 0.52 kΩ.cm^2^, mean ± SEM; *p* = 0.93; Table S1). The barrier was retained during CdCl_2_ exposures of at least seven days, over which time the TEER increased in the cadmium-exposed culture to 1.8-fold over control. Analysis of cell lysates by inductively coupled plasma optical emission spectroscopy (ICP-OES) revealed an intracellular cadmium concentration of 0.94 μM in lysates from cadmium-exposed cultures compared to 0.08 μM for control cultures.

### 2.2. Baseline and Cadmium-Induced MT Transcription in NHU Cells

**Figure 2:**
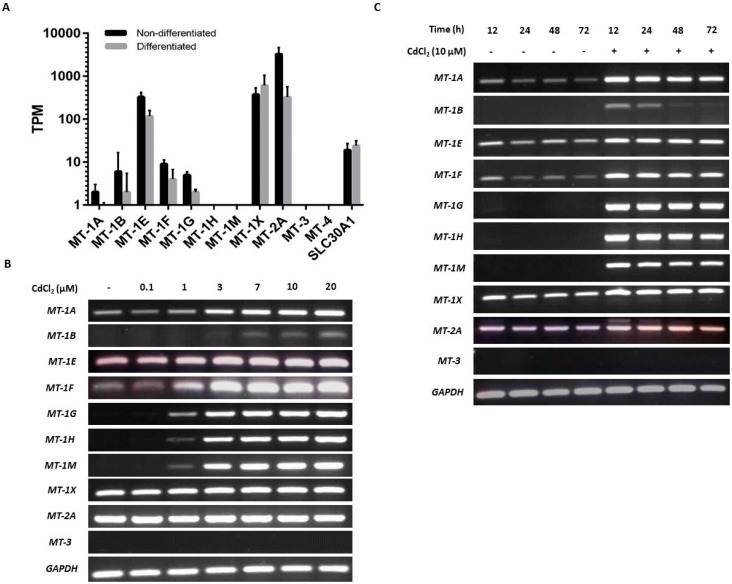
Baseline and cadmium-induced MT transcript expression by NHU cells in vitro. (**A**) Next-generation sequencing data showing baseline MT isoform transcription in nondifferentiated and differentiated NHU cells (*n* = 3 independent cell lines; standard deviation is shown). (**B**,**C**) MT gene expression in NHU cells assessed by RT-PCR. The total cDNA input was 1 µg and PCR reaction products were removed after 25 cycles; *GAPDH* was included as input control. See Table 1 for primer sequences and product sizes. Note that medium was changed at time T = 0 only and there was no renewal of cadmium over the period. The figure shows results representative of *n* = 3 independent NHU cell lines. Additional PCR controls included genomic DNA as a positive control and a no-template (H_2_O) negative control; RT negative samples confirmed absence of genomic contamination. In (**B**), the result of exposing nondifferentiated NHU cells to different concentrations of cadmium (0–20 μM) for 72 h on MT gene expression is shown. In (**C**), MT gene expression is shown in differentiated NHU cell cultures following exposure to 10 µM CdCl_2_ for up to 72 h.

NHU cells maintained in culture in nondifferentiated and differentiated states were examined for baseline expression of MT genes. Analysis by mRNA-seq of nondifferentiated NHU cells revealed high expression of *MT-1E* and *MT-1X* and low expression of *MT-1A*, *MT-1B*, *MT-1F*, and *MT-1G*; there was no detection of *MT-1H* or *MT-1M* transcripts (Figure 2A). *MT-2A* expression was three times greater than all the MT-1 genes combined. No expression was detected for *MT-3* or *MT-4*. In almost all cases where MT gene expression was detected in nondifferentiated NHU cells, the expression was reduced in the differentiated state. This was most striking for *MT-2A* (log_2_FC = 4.2; q = 4.08 × 10^−3^) and *MT-1E* (log_2_FC = 1.5; q = 4.0 × 10^−4^), although between-donor variation prohibited statistical significance for many genes with lower expression. The apparent exception was *MT-1X*, where the average expression increased in the differentiated state. However, this was inconsistent between donors, and the differential expression was nonsignificant. Interestingly, *MT-1L* (which generates a transcript with a premature stop codon [72]) was expressed at similar abundance to *MT-1E* in the nondifferentiated cells, but with a much greater downregulation in the differentiated state (log_2_FC = 5.4; q = 8.4 × 10^−4^). Previous reports of a truncation-rescuing polymorphism [73] was not identified in these donors, so while *MT-1L* is unlikely to form a functional protein, it may play a role in MT-1 transcript regulation. Expression was detected for *SLC30A1* in both nondifferentiated and differentiated states (Figure 2A), but there was no significant differentiation-associated change in expression.

RT-PCR results supported the NGS data, although the variability in transcript detection in nondifferentiated NHU cells indicated a potentially inducible state (Figure 2B). Differentiated NHU cell cultures revealed a more consistent baseline expression of several MT-1 genes, particularly *MT-1X* (Figure 2C).

Exposure to cadmium ions caused a massive induction of all eight MT-1 genes (*MT-1A*, *MT-1B*, *MT-1E*, *MT-1F*, *MT-1G*, *MT-1H*, *MT-1M*, and *MT-1X*) within 12 h of initial exposure, as demonstrated by the RT-PCR results, with expression receding over time (Figure 2B,C). Whereas expression of *MT-1* transcripts was mostly lost after 48 h of continuous cadmium exposure in nondifferentiated cell cultures (Figure 2B with independent cell line repeats in supplementary Figures S1 and S2), MT-1 subfamily transcript expression was still detectable in differentiated cell cultures after 72 h exposure (Figure 2C; with independent repeats in supplementary Figures S3 and S4).

Irrespective of differentiation state and the presence or absence of cadmium, *MT-2A* transcript expression was constitutively high, whilst *MT-3* and *MT-4* transcripts were invariably absent. Based on this, the *MT-2A*, *MT-3*, and *MT-4* genes were not further studied. By contrast, the strong induction of the *MT-1A*, *-1G*, *-1H*, and *-1M* paralogs, which was consistent following cadmium exposure in three independent NHU cell lines, was further investigated for specificity. As the RT-PCR results demonstrated a striking on/off transcriptional response of these MT-1 isoforms to cadmium exposure, it was decided to continue with this approach, as quantitative PCR would not have added anything to the data.

### 2.3. Specificity of Cadmium-Induced MT Transcription

NHU cell cultures were exposed to a variety of candidate MT inducers identified from the literature.

**Figure 3:**
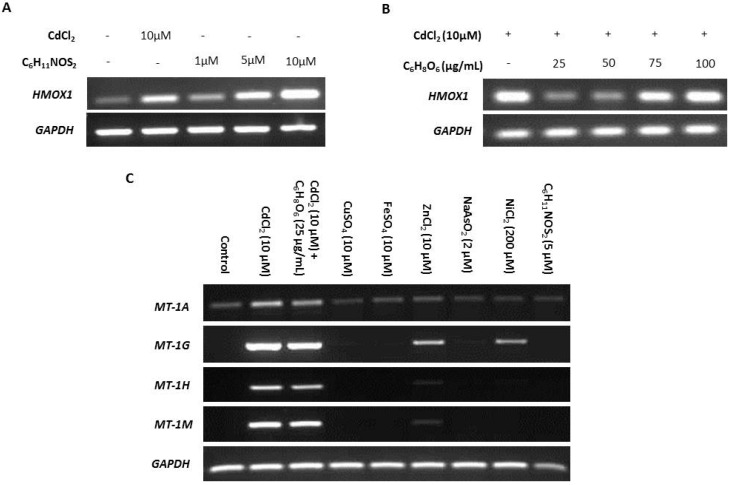
Specificity of metallothionein (MT) transcription induction by cadmium. MT gene expression was assessed by RT-PCR in NHU cells exposed to a variety of candidate inducers. The total cDNA input was 1 µg and PCR reaction products were removed after 25 cycles; *GAPDH* was included as input control. Note that medium was changed at time T = 0 only and there was no renewal of treatments over the period. Additional PCR controls included genomic DNA as a positive control and a no-template (H_2_O) negative control; RT negative samples confirmed absence of genomic contamination. In (**A**), nondifferentiated NHU cell cultures were treated with a range of concentrations of sulforaphane (C_6_H_11_NOS_2_) for 12 h, and the effect on transcription of the ROS-sensitive gene *HMOX1* assessed in comparison to exposure to 10 µM CdCl_2_ (*n* = 1 independent cell line). The concentration of C_6_H_11_NOS_2_ that induced transcription of *HMOX1* to a comparable extent to cadmium was selected. In (**B**), nondifferentiated NHU cell cultures were treated with a range of concentrations of ascorbic acid (C_6_H_8_O_6_) for 12 h in combination with 10 µM CdCl_2_ and the effect on *HMOX1* transcription assessed. The concentration of C_6_H_8_O_6_ that caused the biggest decrease in *HMOX1* transcription was selected. In (**C**), NHU cell cultures (*n* = 2 independent cell lines) were exposed to a range of candidate regulators and transcript expression was assessed for the MT-1 genes shown above to be most sensitive to cadmium induction (from Figure 2B,C). Candidate inducers tested were cadmium (10 µM CdCl_2_), cadmium combined with ascorbic acid (10 µM CdCl_2_ + 25 µg/mL C_6_H_8_O_6_), copper (10 µM CuSO_4_), iron (10 µM FeSO_4_), zinc (10 µM ZnCl_2_), arsenite (2 µM NaAsO_2_), nickel (200 µM NiCl_2_), and sulforaphane (5 µM C_6_H_11_NOS_2_). Essential metals were applied at equivalent concentrations to cadmium. Arsenite and nickel were both used at their highest noncytotoxic concentrations based on initial titration experiments (not shown).

ROS is reported as a by-product of cadmium exposure [59], and therefore we sought to determine the effects of ROS on transcription of MT-1 genes. The ROS-inducing agent sulforaphane (C_6_H_11_NOS_2_) and ROS-inhibitor ascorbic acid (C_6_H_8_O_6_) were titrated against transcription of the ROS-sensitive heme oxygenase-1 (*HMOX1*) gene [74,75] to infer intracellular ROS activity (Figure 3A,B, respectively). Induction of expression of the four MT-1 genes in response to cadmium exposure was unaffected by 25 µg/mL ascorbic acid used to inhibit ROS, suggesting that ROS was not responsible (Figure 3C). This conclusion was supported by the failure of 5 µM sulforaphane to induce MT-1 expression (replicate in supplementary material, Figure S5).

Differential induction of MT-1 paralogs was examined in response to other metal ions, both essential and carcinogenic (replicates in supplementary material, Figures S6 and S7). Both zinc and nickel exposure induced *MT-1G* transcription, whereas *MT-1H* transcript expression was only minimally induced by zinc, and nickel had no effect. *MT-1A* transcription was constitutively low under all conditions apart from cadmium exposure, which increased expression, and *MT-1M* transcription was highly induced by exposure to cadmium alone.

### 2.4. Immunoblotting With Isoform-Specific Antibodies

**Figure 4:**
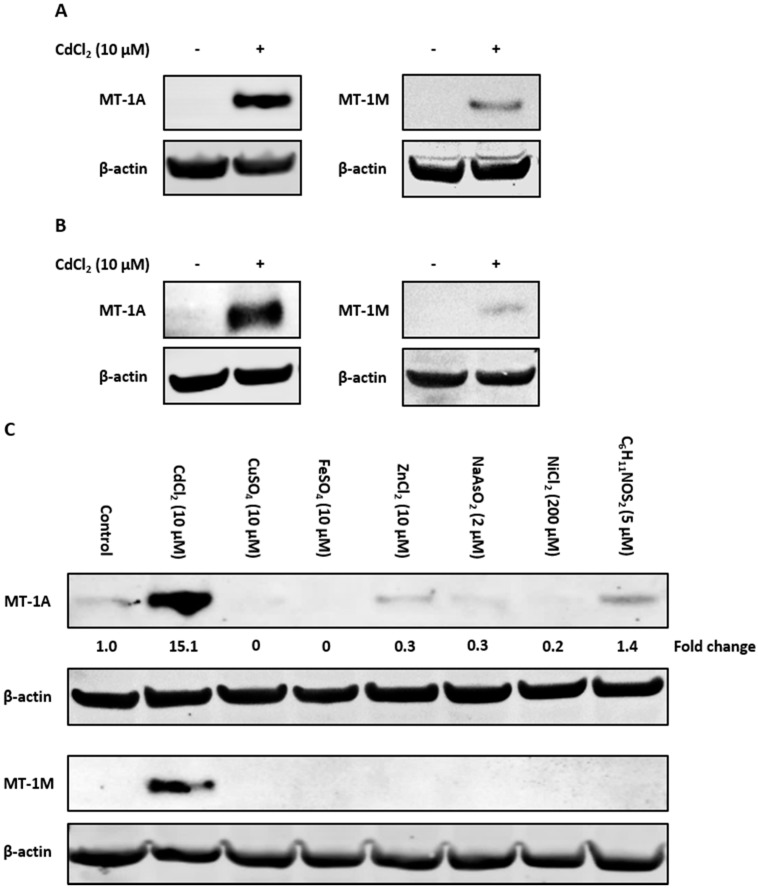
Western blot detection of cadmium-induced MT-1A and MT-1M expression in NHU cells using subtype-specific antibodies. MT-1A and MT-1M protein induction was observed by Western blotting of NHU cells exposed to 10 µM CdCl_2_ for 72 h. Total protein input was 20 µg per track, with β-actin expression used to verify equal protein loading. Figures show western blots probed with MT-1A and MT-1M isoform-specific antibodies in (**A**) nondifferentiated and (**B**) differentiated NHU cell cultures exposed to cadmium for 72 h (representative blots shown from *n* = 3 independent cell lines). In (B), differentiated barrier formation was confirmed by TEER (see Figure 3B). (**C**) Western blot showing specificity of MT-1A and MT-1M protein induction (representative of *n* = 2 independent cell lines tested). Proliferating NHU cells were exposed to a range of potential inducers for 72 h and protein expression assessed. The candidate inducers tested were cadmium (10 µM CdCl_2_), copper (10 µM CuSO_4_), iron (10 µM FeSO_4_), zinc (10 µM ZnCl_2_), arsenite (2 µM NaAsO_2_), nickel (200 µM NiCl_2_), and sulforaphane (5 µM C_6_H_11_NOS_2_). MT-1A protein expression is reported as fold-change relative to unexposed control cells.

To examine if the observed induction of MT-1 gene expression translated to protein, antibodies specific to the MT-1A and MT-1M isoforms were used to perform Western blotting. Control nondifferentiated NHU cells lacked MT-1A and MT-1M protein expression (Figure 4A). Cadmium exposure caused induction of both MT-1A and MT-1M proteins after 72 h (replicate in supplementary material, Figure S8). Both proteins were also induced in cadmium-exposed differentiated NHU cells (Figure 4B; replicated in supplementary material, Figure S9).

The specificity of MT-1A and MT-1M protein induction was examined in response to the wider range of candidate inducers. Western blotting revealed that the MT-1M isoform was induced only by cadmium exposure, supporting the RT-PCR results (Figure 4C; replicated in supplementary material, Figure S10). MT-1A protein expression was highly induced by cadmium exposure, although low protein expression was observed under other conditions. As assessed by densitometry, only cadmium was capable of increasing MT-1A protein expression over control, resulting in a ~6-fold increase in MT-1A protein expression (Figure 4D).

### 2.5. Upregulation of Zinc Transporter-1 (SLC30A1) Transcription in Cadmium-Exposed NHU Cells

**Figure 5:**
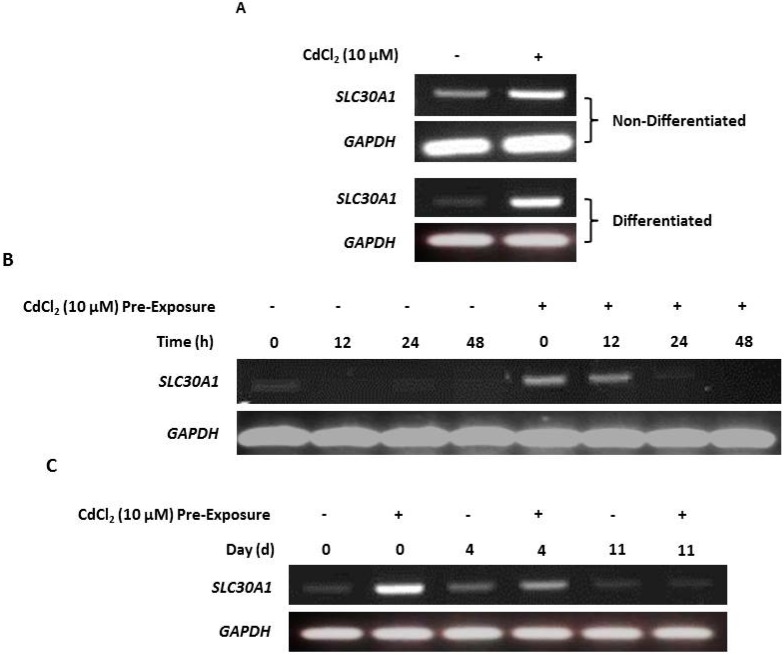
Cadmium induction of zinc transporter-1 (*SLC30A1*) gene transcription. *SLC30A1* gene transcript expression was assessed by RT-PCR, with PCR reaction products removed after 25 cycles. PCR controls included genomic DNA as a positive control and a no-template (H_2_O) negative control. Total cDNA input was 1 µg with *GAPDH* used as an input loading control; RT-ve samples confirmed the absence of genomic contamination. For differentiated NHU cells, differentiation was confirmed by assessment of TEER as >1000 Ω.cm^2^ (see supplementary materials, Table S1). (**A**) NHU cells in nondifferentiated and differentiated states were maintained in control medium or with 10 µM CdCl_2_ for up to 72 h. Note that medium was changed at time T = 0 and there was no renewal of the cadmium over the period. The figure shows representative results from *n* = 3 (nondifferentiated) and *n* = 1 (differentiated) independent NHU cell lines. (**B**) RT-PCR of NHU cells in a nondifferentiated state maintained in standard medium (control) or with 10 µM CdCl_2_ for up to 24 h prior to the experiment (‘pre-exposure’), whereupon cadmium was removed from the medium (time point 0) and culture continued for a further 48 h. (**C**) RT-PCR of NHU cells in a differentiated state maintained in standard medium (control) or with 10 µM CdCl_2_ for up to 3 days prior to the experiment (‘pre-exposure’), whereupon cadmium was removed from the medium (time point 0) and culture continued for up to 11 days.

RT-PCR of both nondifferentiated and differentiated NHU cells revealed that cadmium exposure resulted in increased *SLC30A1* gene transcription compared to unexposed controls (Figure 5A; replicated in supplementary material, Figure S11). After cessation of exposure, *SLC30A1* transcript expression receded over time, and this decrease was observed to occur most rapidly in nondifferentiated (after 24 h; Figure 3B) compared to differentiated (after 11 days; Figure 5C) cell cultures.

## 3. Discussion

To the best of the authors’ knowledge, this is the first study to investigate if cadmium exposure affects urothelial tight barrier function. Previous studies have examined the effect of cadmium exposure on renal [76] and bronchial [77] epithelia, with both studies reporting a decrease or complete collapse of barrier function. By contrast, the results from this study showed that urothelial barrier function was retained and even tightened. This may reflect the type of epithelium being tested, as urothelial cells are known to form one of the tightest epithelial barriers in the human body and thus may be unique in their resistance to cadmium. Although cadmium did not compromise urothelial barrier function, our results found that apical exposure induced expression of MT-1 isoforms in underlying NHU cells and using ICP-OES, cadmium was directly detected in differentiated urothelial cell sheets. Taken together, these results indicate that the urothelium remains intact and may even mount a protective response to cadmium in terms of tight junction tightening, yet the heavy metal is still able to penetrate the urothelial barrier.

Also for the first time, we describe specific MT gene expression by normal human urothelium. Our results are consistent with previous reports in other tissues that MT-1 is the inducible subfamily [22,40,78] whereas *MT-2A* is the most widely expressed isoform, accounting for up to 50 % total MT expression in humans [6,79]. In agreement with the consensus that MT-3 is neural-restricted [80], we found no baseline expression and no agents that could induce *MT-3* transcription in urothelial cells. As an anomaly, one group has previously suggested a link between heavy metal exposure, MT-3 induction, and urothelial carcinogenesis based on high MT-3 expression in malignant bladder cancer [81]. This group further supported their findings using in vitro studies in cadmium-transformed UROtsa cells [82,83]. However, we were not able to replicate those findings in our normal cell system.

Of the MT-1 gene family, transcription of *MT-1F* and *MT-1G* has previously been described as constitutive in human umbilical vein epithelial cells (HUVEC) under baseline conditions [42]. Both these genes exhibited low basal but inducible expression in NHU cells, therefore it cannot be certain whether the expression differences between tissues are constitutive, or perhaps due to inducers present in the culture medium. Whereas we found the majority of MT-1 gene paralogs to be inducible by cadmium exposure, transcript expression was transient, presumably due to sequestration leaving fewer free cadmium ions to maintain induction. By contrast, expression of MT-1 proteins was more stable and in line with the sequestering nature of the formed MT-cadmium complex. Previously, MT-1 protein isoform detection has been performed using mass spectrometry [41], but the demonstrated availability of specific antibodies now opens the door to detection using immunochemical approaches.

Exposure of proliferating NHU cell cultures to a range of divalent metal ions revealed differential and inducer-specific induction of MT-1 isoforms. For example, zinc ions induced strong transcription of *MT-1G*, but not *MT-1A* genes, whereas nickel ions caused *MT-1G* expression exclusively. Inducer-specific expression of MT-1 genes has been noted in other cell types [34,44,45,46,47], supporting the hypothesis that individual isoforms have very selective metal ion sequestering functions [5,6,22,39,40,84]. Western blotting revealed that cadmium was the most potent inducer of MT-1A protein expression, whereas MT-1M protein expression was highly specific to cadmium, revealing the potential of both as biomarkers of cadmium exposure. Neither arsenic nor nickel induced MT-1A or MT-1M protein expression, further demonstrating the specificity of MT-1 isoforms to differentiate between different nongenotoxic carcinogenic metals [85,86].

Alongside a pathophysiological role in sequestering carcinogenic metals, the MTs are considered to contribute to the normal homeostasis of zinc, which as a cofactor involved in many key cellular processes [56] is under tight control. MT sequestration and zinc transporter efflux coordinated by a common transcription factor *MTF-1* [87] is thought to regulate the availability of zinc [88,89,90,91]. Changes in intracellular zinc concentration have been associated with tumor growth and progression [92]. The displacement of zinc from the proteome by cadmium may affect the intracellular concentration and/or availability of zinc ions and can substitute and destabilize the functional sites of zinc-containing proteins, such as zinc-finger transcription factors, changing the character and/or rendering them nonfunctional [54,55].

Preliminary investigation into the effect of cadmium exposure on zinc homeostasis in normal urothelial cells revealed an upregulation of the zinc transporter-1 (*SLC30A1*) gene transcript in nondifferentiated NHU cells. *SLC30A1* upregulation was also observed in cadmium-exposed differentiated NHU cell sheets possessing a functional barrier, further suggesting that cadmium can penetrate an intact urothelial barrier. Our results agree with a previous study using the human hepatic HepG2 cell line, which demonstrated increased *SLC30A1* protein expression and localization at the cell membrane after acute cadmium exposure [93]. A later study showed cadmium exposure resulted in a 93% increase in the intracellular labile zinc concentration, suggesting a large displacement of zinc ions from the proteome, possibly due to substitution by cadmium [94].

MT expression is often seen as a ‘double-edged sword’, as on the one hand it functions to protect the cell, but by the same mechanisms can also facilitate malignant events [6,22,95,96]. MTs may contribute to cell survival by increasing resistance to ROS-induced apoptosis [97] and increasing cellular proliferation [32]. Cadmium exposure can result in inhibition of DNA repair [98], which coupled with increased cellular protection via cadmium-induced MT expression, increases the probability of deleterious cells surviving and passing on defects to their progeny [96]. The ability of MT to counteract ROS could also play a role in chemotherapy resistance, and high expression of MT has been correlated with treatment resistance in bladder cancer [99,100]. Specifically, after radical surgery and adjuvant chemotherapy 100% patients with high tumor MT expression progressed within nine months, whereas in patients with low MT expression only 65% had progressed after five years [101].

Our study supports a concordant induction of MT isoforms and *SLC30A1* transcription in response to cadmium exposure. Whereas this does not directly contradict hypotheses that cadmium exposure increases cellular zinc concentration or that cellular zinc homeostasis is maintained through the cooperative regulation of MT and zinc transporters [39], it does proffer a more direct relationship with cadmium responsible for regulating *SLC30A1* expression. This may reveal new insight as to the role of cadmium in (bladder) cancer, where previously reported *SLC30A1* and MT changes may reflect increased concentrations of intracellular cadmium rather than zinc. Our demonstration of differential MT-1 gene paralog induction by zinc and cadmium should help design future (e.g., knockout) experiments to clarify the respective roles. Further experiments might also directly quantify intracellular zinc in NHU cells after cadmium exposure and determine the consequences of cadmium on zinc homeostasis and dysregulated cadmium-substituted proteins.

## 4. Materials and Methods

### 4.1. NHU Cell Culture and Exposure to Cadmium and Other Agents

Normal human urothelial (NHU) cells were obtained from the ureter/renal pelvis of patients undergoing urological surgery, and maintained in vitro as nonimmortalized cell lines, as detailed elsewhere [57,102]. For routine culture, NHU cells were grown as adherent monolayers on Primaria™ plasticware (BD Biosciences, Wokingham, UK) in low calcium [0.09 mM] keratinocyte serum-free medium (KSFM) containing bovine pituitary extract and recombinant epidermal growth factor (Fisher Scientific UK Ltd, Loughborough, UK) supplemented with 30 ng/mL cholera toxin (KSFMc). NHU cell lines were subcultured by trypsinization at just-confluence and used in experiments between passages 3–5.

For cadmium exposure, medium was replaced with fresh medium containing 10 µM cadmium chloride (CdCl_2_). This concentration was selected after preliminary titration for toxicity (Figure 1A,B). For other treatment agents, concentrations were selected following initial titration and target gene expression assessment (Figure 3A,B).

Nonimmortalized NHU cell lines retain the capacity to differentiate to form a functional tight barrier epithelium [58]. Differentiation was induced by switching NHU cells into medium supplemented with 5% adult bovine serum for 5 days before subculture onto semipermeable ThinCert™ (Greiner Bio-One Ltd., Stonehouse, UK) membranes with 0.4-µm pore size. After 24 h, the exogenous calcium (Ca^2+^) concentration was increased to 2 mM (near physiological) and cultures were maintained for a further 7–9 days to develop a tight barrier. For cadmium exposure of differentiated NHU cell cultures, medium was removed from the apical chamber after establishment of a barrier >1000 Ω.cm^2^ (see below) and replaced with fresh medium supplemented with 10 µM CdCl_2_, in order to mimic apical exposure. RNA and protein were then harvested from these membranes for further analysis.

### 4.2. Measurement of Transepithelial Electrical Resistance

The barrier function of differentiated NHU cell sheets was assessed in triplicate cultures by measuring the transepithelial electrical resistance (TEER) using an EVOM™ voltohmmeter (World Precision Instruments, Hertfordshire, UK), as described [103]. A blank (no cell) membrane measurement was subtracted from each TEER reading.

### 4.3. MT Transcript Abundance Quantification by Next-Generation Sequencing

mRNA-seq data for three donor-matched NHU cultured nondifferentiated and differentiated samples were previously generated by our group [104]. Sequencing reads were ‘pseudoaligned’ to the Ensembl v.91 human transcriptome (GRCh38.p10) using kallisto v0.44.0 [105] and relative gene abundance was calculated as transcripts per million (TPM) following gene-level aggregation with tximport v1.8.0 [106]. Differentiation-associated expression changes in the MT gene family were detected by a differential expression analysis conducted by sleuth [107] accounting for matched-donor samples. Differentially-expressed genes are reported with their log_2_ transformed fold change (log_2_FC) and ‘q’ value (Benjamini–Hochberg correction).

### 4.4. Reverse Transcriptase-Polymerase Chain Reaction (RT-PCR)

RT-PCR was performed to observe actual patterns of MT isoform induction (rather than relative change). RNA was extracted from cells using TRIzol^®^ (Fisher Scientific UK Ltd., Loughborough, UK) and treated with a DNA-free™ kit (Ambion, supplied by Fisher Scientific UK Ltd., Loughborough, UK). cDNA synthesis was performed on 1 µg RNA with random hexamers and the SuperScript^®^II First-Strand Synthesis System (Fisher Scientific UK Ltd., Loughborough, UK). PCR primers were designed specifically to detect all known splice variants for each MT-1 isoform gene, with GAPDH used as the internal transcript control [108]. Primer sequences and optimized PCR conditions are provided in Table 1. PCR was carried out in a T100 thermal cycler (Bio-Rad Services UK Ltd., Hemel Hempstead Hertfordshire, UK) using 25 reaction cycles. Controls consisted of genomic DNA (gDNA as template positive control), water (no template control), and no reverse transcriptase (gDNA negative control). PCR products were separated on 2 % (*w*/*v*) agarose gels, stained using SYBR^®^ Safe DNA gel stain (Invitrogen supplied by Fisher Scientific UK Ltd., Loughborough, UK) and visualized on a Gene Genius Gel Imaging System (Syngene, Cambridge, UK).

### 4.5. Western Blotting

NHU cell cultures were lysed into electrophoresis sample buffer containing protease inhibitors and sonicated. Twenty micrograms of protein was resolved on 4–12% Bis-Tris NuPage™ polyacrylamide gels (Invitrogen) in 2-(N-morphilino) ethanesulfonic acid (MES) buffer and electro-transferred onto polyvinylfluoride membranes (Millipore). Membranes were blocked with Odyssey^®^ blocking buffer (LI-COR Biotechnology OK Ltd., Cambridge, UK), incubated with primary antibodies for 16 h at 4 °C and bound antibody detected using Alexa Fluor^®^ 680-conjugated anti-mouse secondary antibody (Invitrogen, Invitrogen supplied by Fisher Scientific UK Ltd., Loughborough, UK) or an IRDye 800-conjugated anti-rabbit secondary antibody (Tebu-Bio, Peterborough, UK). Antibody binding was visualized using an Odyssey^®^ Sa Infrared Imaging System (LI-COR^®^). Protein quantification was performed using Odyssey^®^ Image Studio™ software v5.0 (LI-COR^®^). Details of antibodies are given Table 2 and Table 3.

## 5. Conclusions

MT-1 isoform expression has been characterized in normal human urothelium for the first time, and a unique expression profile described with the use of isoform-specific antibodies. Individual MT-1 genes demonstrated inducer-specific expression and two paralogs with cadmium-specific or -selective induction were identified as candidate biomarkers of cadmium exposure. With the potential for cadmium to interfere and substitute in the homeostatic regulation of zinc, new approaches are proposed for understanding cadmium-induced nongenotoxic carcinogenesis.

## Figures and Tables

**Table 1:** Details of primers used for experiments.

Target Gene	Forward or Reverse	Sequence (5′–3′)	Product Size (bp)
***GAPDH***	Forward	CAAGGTCATCCATGACAACTTTG	90
***GAPDH***	Reverse	GGGCCATCCACAGTCTTCTG	90
***HMOX1***	Forward	CCAGCAACAAAGTGCAAGATTC	102
***HMOX1***	Reverse	GTGTAAGGACCCATCGGAGAAG	102
***MT-1A***	Forward	CTCGAAATGGACCCCAACT	219
***MT-1A***	Reverse	ATATCTTCGAGCAGGGCTGTC	219
***MT-1B***	Forward	GGAACTCCAGGCTTGTCTTGG	77
***MT-1B***	Reverse	TTGCAGGAGGTACATTTG	77
***MT-1E***	Forward	TGCGCCGGCTCCTGCAAGTC	118
***MT-1E***	Reverse	ATGCCCCTTTGCAGACGCAGC	118
***MT-1F***	Forward	CCTGCACCTGCGCTGGTTCC	110
***MT-1F***	Reverse	ACAGCCCTGGGCACACTTGC	110
***MT-1G***	Forward	CTTCTCGCTTGGGAACTCTA	309
***MT-1G***	Reverse	AGGGGTCAAGATTGTAGCAAA	309
***MT-1H***	Forward	CCTCTTCTCTTCTCGCTTGG	317
***MT-1H***	Reverse	GCAAATGAGTCGGAGTTGTAG	317
***MT-1M***	Forward	CTAGCAGTCGCTCCATTTATCG	180
***MT-1M***	Reverse	CAGCTGCAGTTCTCCAACGT	180
***MT-1X***	Forward	GGACCCAACTGCTCCTGCTC	151
***MT-1X***	Reverse	TTTGCAGATGCAGCCCTGGGC	151
***MT-2A***	Forward	CCGACTCTAGCCGCCTCTT	259
***MT-2A***	Reverse	GTGGAAGTCGCGTTCTTTACA	259
***MT-3***	Forward	AGTGCGAGGGATGCAAATG	98
***MT-3***	Reverse	GCCTTTGCACACACAGTCCTT	98
***SLC30A1***	Forward	GCATCAGTTTATGAGGCTGGTCCT	352
***SLC30A1***	Reverse	CAGGCTGAATGGTAGTAGCGTGAA	352

**Table 2:** Details of primary antibodies used for experiments.

Antigen	Clone	Host	Supplier	Dilution	Molecular Weight (kDa)
**Beta-actin**	A5441	Mouse	Sigma Aldrich	1:10 000 (WB)	42
**MT-1A**	B01P	Mouse	Abnova	1:750 (WB)	6
**MT-1M**	17281-AP	Rabbit	ProteinTech	1 µg/mL (WB)	6

**Table 3:** Details of secondary antibodies used for experiments.

Antigen	Conjugate	Host	Supplier	Application
**Anti-mouse IgG**	Alexa 680	Goat	Life Technologies	WB
**Anti-rabbit IgG**	Alexa 800	Goat	Life Technologies	WB

## Supplementary Materials

Supplementary materials can be found at https://www.mdpi.com/1422-0067/20/6/1344/s1.

## Abbreviations

HMOX1	Heme oxygenase-1
MT	Metallothionein
NHU	Normal human urothelial
SLC30A1	Solute carrier family A member 1
TEER	Trans-epithelial electrical resistance
