# Supplementary material for: Specificity of the Metallothionein-1 Response by Cadmium-Exposed Normal Human Urothelial Cells
Source: Int J Mol Sci. 2019 Mar 17;20(6):1344. doi: 10.3390/ijms20061344 (PMC6471910; doi:10.3390/ijms20061344)
Supplement: Supplementary file 1 [file ijms-20-01344-s001.pdf]

Supplementary data for "Specificity of the Metallothionein-1 Response by Cadmium-Exposed Human Urothelial Cells"

Rhiannon V. McNeill, Andrew S. Mason, Mark E. Hodson, James W.F. Catto, Jennifer Southgate.

Supplementary Table S1: Transepithelial electrical resistance (TEER) readings from different NHU cell lines used for experiments. Each reading is an average of three technical replicates. A TEER reading >0.5 kΩ.cm^2 is considered to reflect a functional urothelial barrier.

Cell Line	Figure	Time Point	TEER (kΩ.cm^2)	
			Control	Cadmium
Y1456	1B, Suppl.	12h	3.54	4.88
		24h	3.58	3.96
		48h	4.63	2.14
		72h	3.78	4.09
Y1493	Suppl.	24h	2.00	2.65
		48h	3.20	2.99
		72h	2.27	2.30
Y1426	4C, Suppl.	72h	3.33	4.43

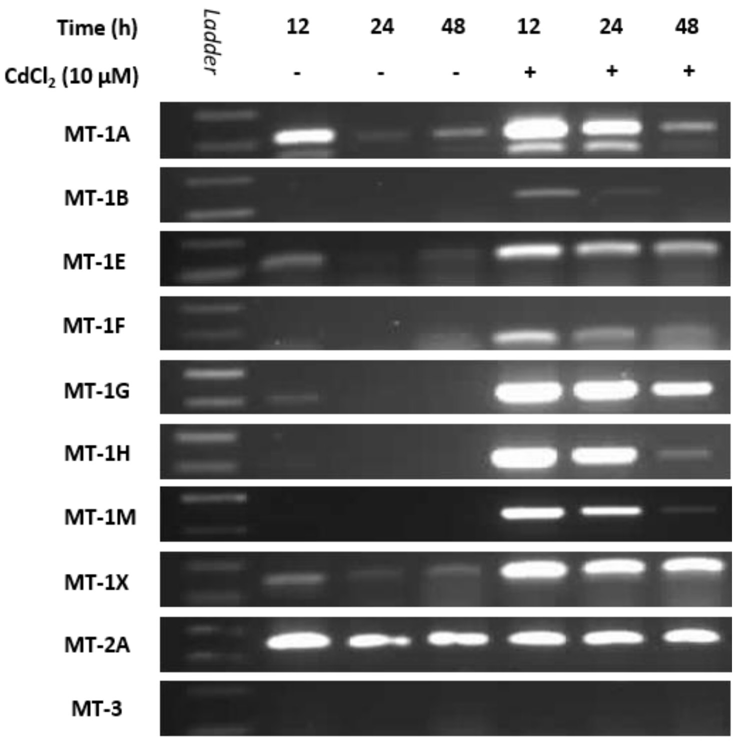

Supplementary Figure 1. RT-PCR showing MT isoform transcription in proliferating NHU cells exposed to cadmium (experimental replicate from Figure 2B). Nondifferentiated NHU cells were exposed to 10 μM $CdCl_2$ for up to 48 h. The total cDNA input was 1 μg and PCR reaction products were removed after 25 cycles.

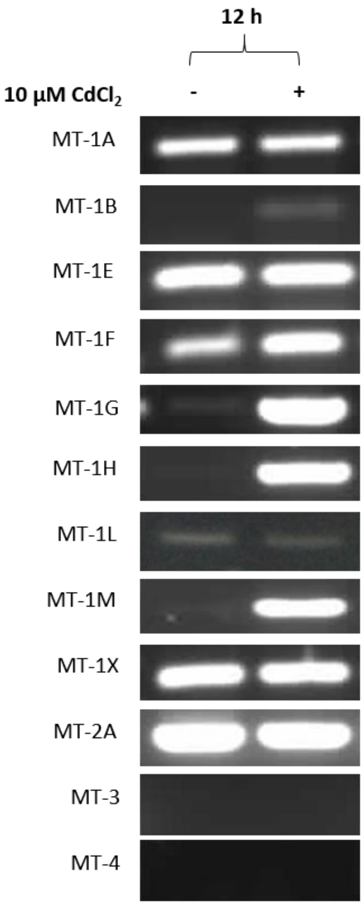

Supplementary Figure 2. RT-PCR showing MT isoform transcription in proliferating NHU cells exposed to cadmium (experimental replicate from Figure 2B). Nondifferentiated NHU cells were exposed to 10 μM $CdCl_2$ for up to 12 h. The total cDNA input was 1 μg and PCR reaction products were removed after 25 cycles.

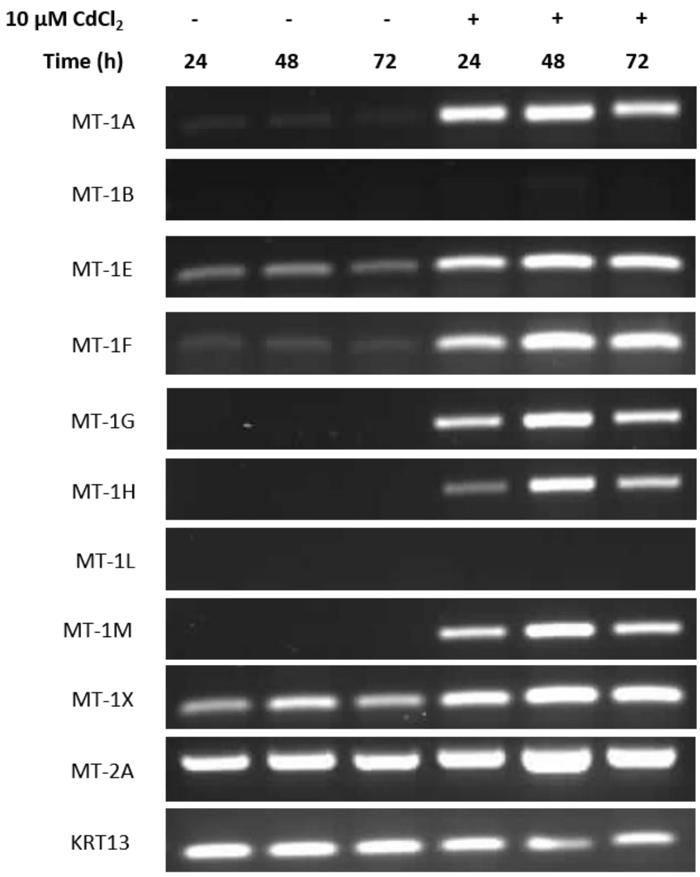

Supplementary Figure 3. RT-PCR showing MT isoform transcription in cadmium-exposed differentiated NHU cell sheets that demonstrated a functional barrier (experimental replicate from Figure 2C). NHU cells were differentiated and exposed to 10 μM $CdCl_2$ for up to 72 h. Differentiation was confirmed using TEER readings and expression of KRT13. The total cDNA input was 1 μg and PCR reaction products were removed after 25 cycles.

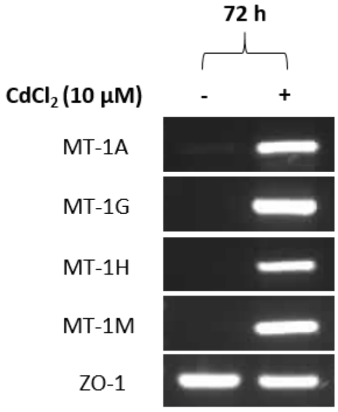

Supplementary Figure 4. RT-PCR showing MT isoform transcription in cadmium-exposed differentiated NHU cell sheets that demonstrated a functional barrier (experimental replicate from Figure 2C). NHU cells were differentiated and exposed to 10 μM $CdCl_2$ for up to 72 h. Differentiation was confirmed using TEER readings and expression of ZO-1. The total cDNA input was 1 μg and PCR reaction products were removed after 25 cycles.

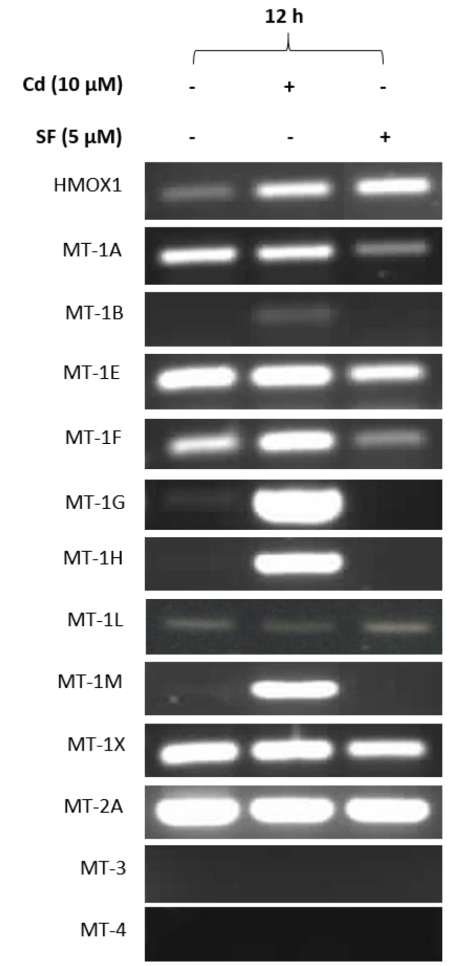

Supplementary Figure 5. RT-PCR showing the effects of ROS on MT isoform transcription in NHU cells (experimental replicate from Figure 3C). The chemical sulforaphane ($C_6H_{11}NOS_2$) was used to induce ROS, having been titrated to a concentration that mimicked the levels of cadmium-induced ROS. Nondifferentiated NHU cells were treated with either 10 μM $CdCl_2$ or 5 μM $C_6H_{11}NOS_2$ for 12 h. The total cDNA input was 1 μg and PCR reaction products were removed after 25 cycles.

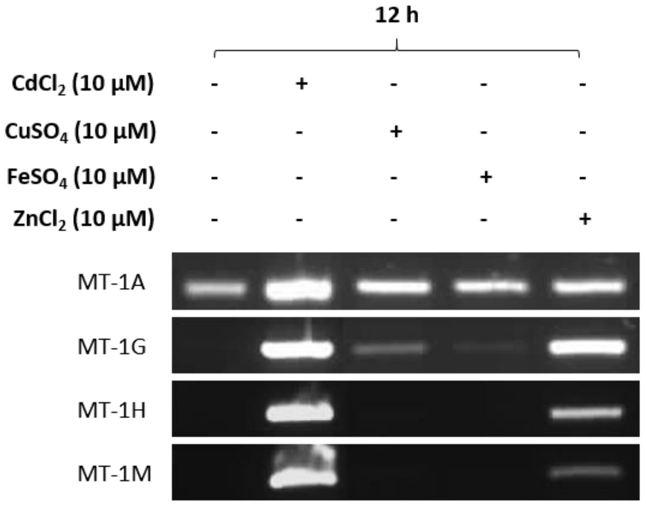

Supplementary Figure 6. RT-PCR showing the effects of essential metal exposure on MT-1 isoform transcription in NHU cells (experimental replicate from Figure 3C). Nondifferentiated NHU cells were exposed to either 10 μM $CdCl_2$, 10 μM $CuSO_4$, 10 μM $FeSO_4$, or 10 μM $ZnCl_2$ for 12 h. The total cDNA input was 1 μg and PCR reaction products were removed after 25 cycles.

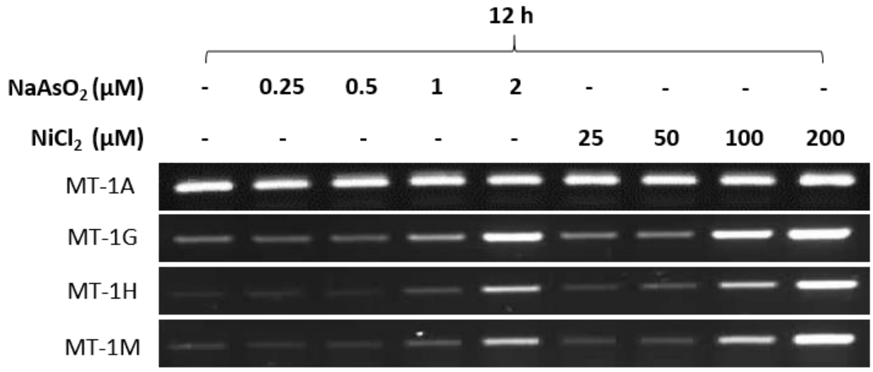

Supplementary Figure 7. RT-PCR showing the effects of exposure to the carcinogenic metals arsenite and nickel on MT-1 isoform transcription in NHU cells (experimental replicate from Figure 3C). Nondifferentiated NHU cells were exposed to a range of concentrations of arsenite (0.25–2 μM; NaAsO2) and nickel (25–200 μM; $NiCl_2$) for 12 h. The total cDNA input was 1 μg and PCR reaction products were removed after 25 cycles.

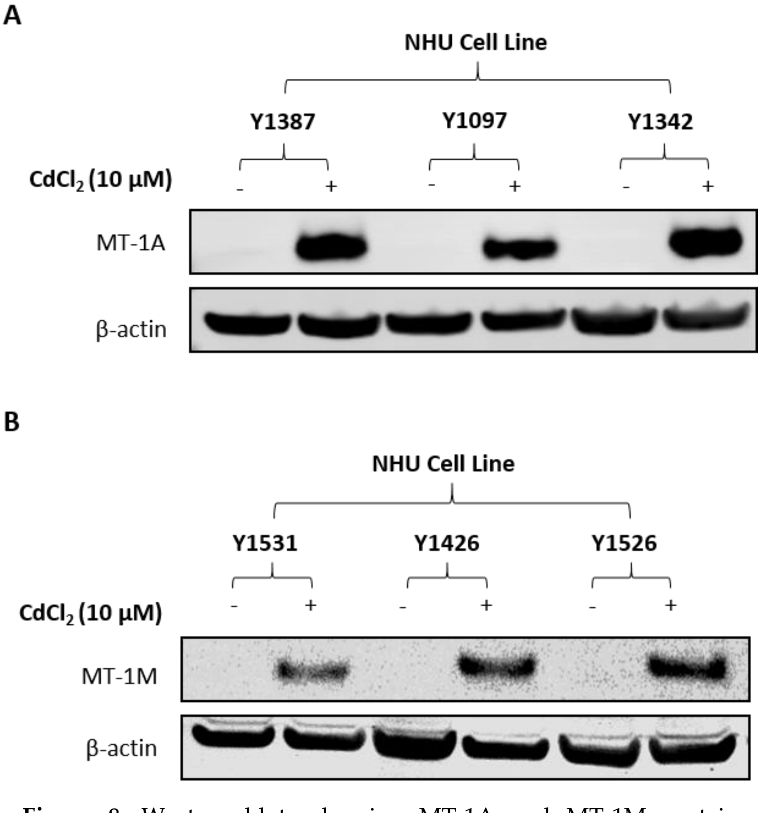

Supplementary Figure 8. Western blots showing MT-1A and MT-1M protein expression in nondifferentiated NHU cells exposed to cadmium (experimental replicate from Figure 4A). Nondifferentiated NHU cells (n = 3) were exposed to 10 μM $CdCl_2$ for 72 h and protein expression of (A) MT-1A and (B) MT-1M determined using novel, isoform-specific antibodies. β-actin protein expression was used as a loading control.

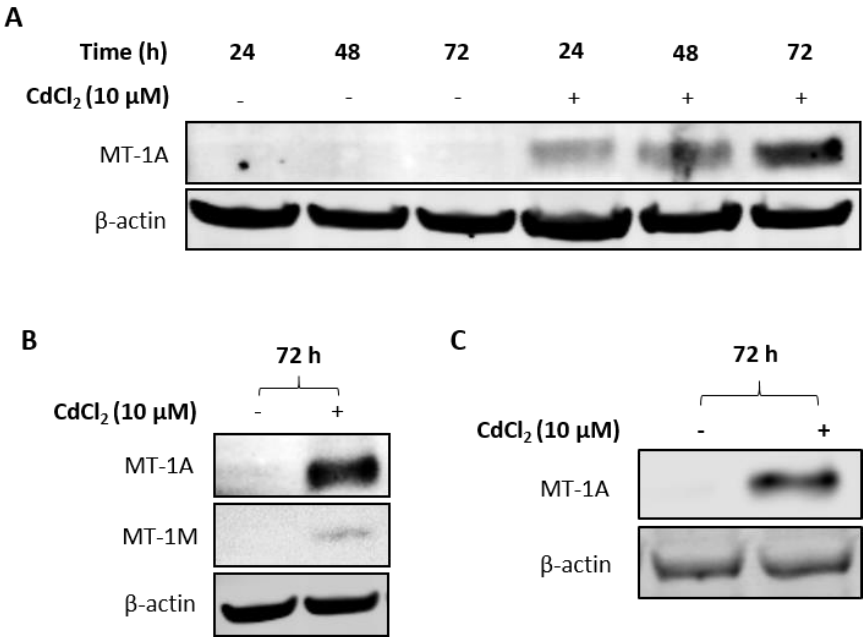

Supplementary Figure 9. Western blots demonstrating MT-1A and 1M protein expression in differentiated NHU cells with functional barriers that were exposed to cadmium (experimental replicate from Figure 4B). NHU cells were stimulated to differentiate and form a functional barrier, before exposure to 10 µM $CdCl_2$. (A) MT-1A protein expression was assessed at multiple time-points to ensure exposure time was adequate for protein translation in differentiated NHU cells. (B) Western blots showing MT-1A and MT-1M protein expression and (C) MT-1A protein expression in differentiated NHU cells exposed to cadmium for 72 h. β-actin protein expression was used as a loading control.

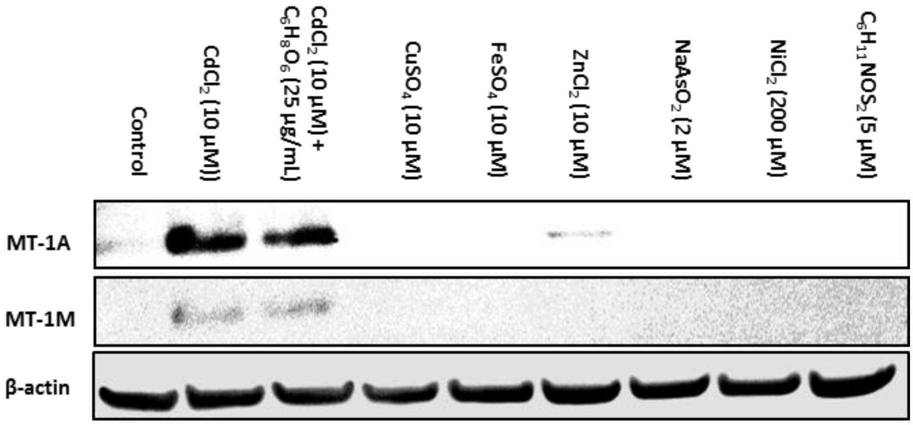

Supplementary Figure 10. Western blot showing the specificity of cadmium-induced MT-1A and MT-1M isoform protein expression in NHU cells (experimental replicate from Figure 4C). Nondifferentiated NHU cells were exposed to a range of potential inducers for 72 h. Candidate inducers were cadmium (10 µM $CdCl_2$), copper (10 µM $CuSO_4$), iron (10 µM $FeSO_4$), zinc (10 µM $ZnCl_2$), arsenite (2 µM $NaAsO_2$), nickel (200 µM $NiCl_2$), and sulforaphane (5 µM $C_6H_{11}NOS_2$). Cadmium in combination with ascorbic acid (25 µg/mL $C_6H_8O_6$) was also included, to support the RT-PCR data demonstrating that inhibition of cadmium-induced ROS did not inhibit cadmium-induced MT expression (Figure 3C). β-actin protein expression was used as a loading control.

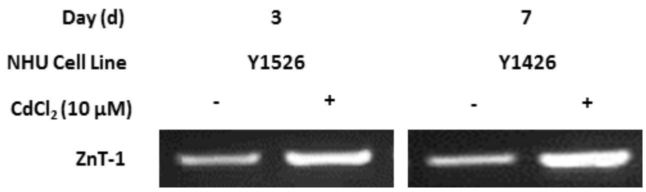

Supplementary Figure 11. Effect of cadmium exposure on zinc transporter-1 (SLC30A1) transcription (experimental replicate from Figure 5B). RT-PCR of SLC30A1 gene transcription in nondifferentiated NHU cells exposed to 10 μM $CdCl_2$ for 3 or 7 days. Note that medium was changed at time T = 0 and that for 3 day exposure there was no renewal of the cadmium by medium change over the period. For 7 day exposure, cadmium-containing medium was renewed on day 4. The total cDNA input was 1 μg and PCR reaction products were removed after 25 cycles.
